# Supplementary material for: A Survey of Telemedicine Use by Doctors in District Hospitals in KwaZulu-Natal, South Africa
Source: Int J Environ Res Public Health. 2022 Oct 11;19(20):13029. doi: 10.3390/ijerph192013029 (PMC9602563; doi:10.3390/ijerph192013029)
Supplement: Supplementary file 1 [file ijerph-19-13029-s001.zip › ijerph-1898892-supplementary.pdf]

Supplementary Materials: – Questionnaire

INFORMAL TELEMEDICINE SERVICES IN KZ-N HOSPITALS

Telemedicine is the use of information and communication technologies to provide healthcare over distance. Some examples that you may be familiar with are the use of videoconferencing for teledermatology or education, taking photographs and sending them by phone when referring a burns case to IALCH, referring cases by email or using instant messaging such as WhatsApp to refer a case or seek advice from a colleague.

AIMS OF THE STUDY

An informal and unplanned teledermatology service has evolved between rural doctors and the dermatologists at the Medical School using the smartphone instant messaging app, WhatsApp. We want to better understand how this service began, why some people who know about it may not be using it and whether those who are not currently aware of it would or would not use it in in the future. We are also trying to determine what the referral load would be if this were established as a Province wide service. This information will assist the Dept of Dermatology in organizing their staff to meet the need.

At the same time we are exploring whether similar unplanned and undocumented telemedicine activities exist in other disciplines and what technologies or apps are being used. Examples being photographs of ECGs or X-Rays sent to a colleague for an opinion or advice by smartphone using instant messaging, email or social media. Also, whether you feel there is need for services to be developed in other disciplines and if you would use them.

We need to understand what your concerns are or might be about using telemedicine services, especially instant messaging eg WhatsApp.

Questionnaire

I have read the consent form and consent to participate in this survey. ☐

Section 1

Demographic information

1. Your age: 25 – 30 ☐ 31 – 40 ☐ 41 – 50 ☐ 50 – 60 ☐ >60 ☐

2. Gender: Male ☐ Female ☐

3. Years since medical graduation

   1 – 5 ☐ 6 – 10 ☐ 11 – 20 ☐ 21 – 30 ☐ >30 ☐

4. Additional Post-grad /qualification? (Tick all that apply)

   Certificate ☐ Diploma ☐ Masters ☐ MD/PhD ☐ Specialist ☐

5. What is your speciality?

6. Technology. Do you own any of the following devices. (Tick all that apply)

Smartphone	Yes	No
Tablet PC	Yes	No
Computer	Yes	No

7. Technology - connectivity (Tick all that apply)

Do you have access to a computer at work?	Yes	No
Do you have Internet access at work?	Yes	No
Do you have wireless access at work?	Yes	No

Section 2

Teledermatology by instant messaging

8. Before taking part in this survey, were you aware that doctors in the public sector in KwaZulu-Natal are using WhatsApp and other forms of Instant Messaging to seek advice on the diagnosis and management of dermatological problems by taking photos of the lesions and sending chat messages?

   Yes ☐ No ☐

9. Have you ever used WhatsApp or other instant messaging service for teledermatology?

   Yes ☐ No ☐

   If Yes, please complete questions 9 – 25
   If No, please go to question 26

10. Which instant messaging applications have you used for dermatology? Tick all that apply.

WhatsApp	☐	Facetime	☐	Google chat	☐
Viber	☐	Facebook	☐	Snapchat	☐
Line	☐	Instagram	☐	WeChat	☐
Kik Messenger	☐	Telegram	☐	Skype	☐

Other (Please state) ______________________

11. Who do you send your teledermatology consults to? Tick all that apply.

Dermatologists at the Medical School	☐	Drs at your hospital	☐
Dermatologists in KZN Public Hospitals	☐	Drs at other public hospitals	☐
Dermatologists in private practice	☐	Drs in private practice	☐
Dermatologists in other Provinces	☐	Others	☐

12. Does the WhatsApp or other instant messaging service meet your needs?

   Yes ☐ No ☐

13. How satisfied are you with the Instant messaging teledermatology?

Very dissatisfied	Dissatisfied	Unsure	Satisfied	Very satisfied

14. What has the average response time been to your messages?

<10 min	<30 min	<1 hour	1-3 h	3-6 h	6-12 h	12-24 h	>24 h

15. What is the average response time that you would like to have/

<10 min	<30 min	<1 hour	1-3 h	3-6 h	6-12 h	12-24 h	>24 h

16. Was the information/diagnosis/ management plan/advice provided by the dermatologist helpful?

Always	Mostly	About half the time	Seldom	Never

17. Do you follow the advice given?

Always	Mostly	About half the time	Seldom	Never

18. Do you feel that your patients are satisfied with the teledermatology consultation and process?

Always	Mostly	About half the time	Seldom	Never

19. Do you feel that your patients are satisfied with you taking photographs of their lesions?

Always	Mostly	About half the time	Seldom	Never

20. Did you have any difficulty taking photographs?

Always	Mostly	About half the time	Seldom	Never

21. Did you have any difficulty attaching photographs to your text messages?

Always	Mostly	About half the time	Seldom	Never

22. When sending messages, what form of connectivity did you use? Tick all that apply.

The hospital wireless network	Yes	No
A hospital networked computer	Yes	No
Your own data bundle on your phone or tablet	Yes	No

23. How did you become aware of instant messaging for dermatology?

Thought of it myself	Yes	No
From a colleague at work	Yes	No
From a colleague at another hospital	Yes	No
From the KZ-N DOH	Yes	No
From a dermatologist	Yes	No

24. How did you choose which dermatologist to consult?

From a list of dermatologists provided by the DOH	Yes	No
From a list of dermatologists provided by the Medical School	Yes	No
The dermatologist on call	Yes	No
Personal acquaintance with the dermatologist	Yes	No
On the advice of a colleague	Yes	No
The dermatologist who conducts outreach at our hospital	Yes	No

25. How frequently do you use instant messaging teledermatology?

At least weekly	At least monthly	At least once a quarter	Ad hoc when needed

26. Any additional comments:____

____

____

Section 3

Telemedicine

27. Are you aware of telemedicine services in KZ-N? Yes ☐ No ☐

28. Are you using any telemedicine services? Yes ☐ No ☐

29. If you are aware of telemedicine services but **NOT USING** them, please state why you are not using them? (Tick all that apply.)

Don't know what to do	☐	Takes too long to do	☐
Did not know about it	☐	Not interested	☐
No training in telemedicine	☐	No need	☐
Not required by the DOH	☐	Have concerns about telemedicine	☐
No services available	☐	Absence of telemedicine guidelines	☐
Videoconferencing not available	☐	Legal and ethical concerns	☐
No equipment	☐	No technology support	☐

Other Reasons: ____

____

____

____

30. If you are using telemedicine services what have you used them for and what method did you use? Please tick all that apply.

(VC= videoconferencing; IM = instant messaging eg WhatsApp;

	Yes	VC	email	IM	Social Media	Cell phone calls	Web Site	Other
consultation								
second opinion								
seeking a diagnosis								
management advice								
education								

31. If you are using telemedicine services how frequently do you use them? Please tick all that apply.

	Daily	At least weekly	At least monthly	At least once a quarter	Ad hoc when needed
Videoconferencing					
eMail					
Instant messaging					
Facebook					
Cell Phone					

32. If you using telemedicine, which services do you use it for?

Dermatology		Orthopaedics		Burns	
Radiology		Cardiology		High care	
Psychiatry		ENT		Obstetrics	
Paediatrics		Urology		Rehabilitation	

Other (Please State) ______________________________

33. Are you satisfied with the telemedicine services that you use?

Very dissatisfied	Dissatisfied	Unsure	Satisfied	Very satisfied

Additional comments:______________________________

____

____

34. What form of consent if any do you obtain for: Videoconferenced and store and forward telemedicine, eg instant messaging, emails etc.?

	Videoconferencing		Store &forward	
It is not required	Yes	No	Yes	No
Implied consent	Yes	No	Yes	No
Verbal consent	Yes	No	Yes	No
Written consent	Yes	No	Yes	No

35. If you are using instant messaging for telemedicine, which services do you use it for?

Dermatology		Orthopaedics		Burns	
Radiology		Cardiology		High care	
Psychiatry		ENT		Obstetrics	
Paediatrics		Urology		Rehabilitation	

Other (Please State) ____________________

36. Use of chat groups.

Do you use chat groups for work related activities?	Yes	No
If **No**, do others in your hospital use chat groups for work	Yes	No
If **Yes,** for which of the following activities are they used?		
Administration eg meeting reminders, theatre bookings etc	Yes	No
Seeking advice about a patient	Yes	No
Giving advice to junior staff	Yes	No
Informing colleagues about the status of a patient	Yes	No
Informing colleagues about new admissions/discharges	Yes	No
Informal discussion about a problem	Yes	No
Education/CME	Yes	No

37. If you are **NOT** using telemedicine services, would you like to use a telemedicine service?

Yes ☐ [illegible] ☐ [illegible]decided ☐

38. Whether you are using telemedicine or not, what telemedicine services would you like to have available to you?

Dermatology		Orthopaedics		Burns	
Radiology		Cardiology		High care	
Psychiatry		ENT		Obstetrics	
Paediatrics		Urology		Rehabilitation	
Plastic surgery		Maxillofacial		Dentistry	
Acute medical care		Wound Mx		HIV Mx	

Other, please state:____________________

____

39. If you were to use the instant messaging teledermatology service, how frequently do you think you would use it?

At least weekly	At least monthly	At least once a quarter	Ad hoc when needed

40. If a teleradiology service was available, about how many sets of XRays would you refer on average to a radiologist each week?

None	0-1	2-5	6-10	11-20	>20

41. Are you aware of guidelines for telemedicine practice from any of the following organisations?

The HPCSA	Yes	No
The South African Medical Association	Yes	No
The World Medical Association	Yes	No
The Medical Defence Union	Yes	No
The National Dept of Health	Yes	No

42. What sort of security do you use on your smartphone?

Password/PIN	Yes	No	Don't know
Timed screen locking	Yes	No	Don't know
Fingerprint authentication	Yes	No	Don't know
Disabling or remote wiping	Yes	No	Don't know
Full device encryption	Yes	No	Don't know
Third party lock screen app	Yes	No	Don't know

43. Any other comments?

____

____

____

____

Thank you for your participation.
